# Exploring the Connection Between Nanomaterials and Neurodegenerative Disorders

**DOI:** 10.3390/mi15111382

**Published:** 2024-11-15

**Authors:** Sitansu Sekhar Nanda, Dong Kee Yi

**Affiliations:** Department of Chemistry, Myongji University, Yongin 17058, Republic of Korea; sitansusekhar.nanda@prestigebio.com

**Keywords:** NMs, neurodegenerative, ROS, Alzheimer’s, Huntington’s, Parkinson’s

## Abstract

Drug delivery, tissue engineering, and cell promotion in biomedical fields heavily rely on the use of nanomaterials (NMs). When they penetrate cells, NPs undergo degradation and initiate the generation of reactive oxygen species (ROS) by causing changes in the structures of organelles linked to mitochondria. Inside the cell, the excess production of ROS can initiate a chain reaction, along with the autophagy process that helps maintain ROS balance by discarding unnecessary materials. At present, there is no effective treatment for Alzheimer’s disease (AD), a progressive neurodegenerative disease. The use of NMs for siRNA delivery could become a promising treatment for AD and other CNS disorders. Recent research demonstrates that the use of combined NPs can induce autophagy in cells. This article emphasizes the importance of the shape of siRNA-encapsulated NMs in determining their efficiency in delivering and suppressing gene activity in the central nervous system. Because of its strict selectivity against foreign substances, the blood–brain barrier (BBB) significantly hinders the delivery of therapeutic agents to the brain. Conventional chemotherapeutic drugs are significantly less effective against brain cancers due to this limitation. As a result, NMs have become a promising approach for targeted drug delivery, as they can be modified to carry specific ligands that direct them to their intended targets. This review thoroughly examines the latest breakthroughs in using NMs to deliver bioactive compounds across the BBB, focusing on their use in cancer treatments. The review starts by examining the structure and functions of the BBB and BBTB, and then emphasizes the benefits that NMs offer.

## 1. Introduction

Neurodegenerative diseases (NDs) cause nervous system dysfunction, marked by neuron loss and myelin sheath breakdown [1]. Due to the brain’s intricate structure and operation, identifying the root cause of NDs has proven challenging, hindering progress in treating their symptoms. The blood–brain barrier and glial cells play a crucial role in maintaining an optimal environment for neuronal function [1]. Due to the lack of available early-stage diagnostic tools in medical technology, treating neurodegenerative disorders like Alzheimer’s and Parkinson’s has been a persistent challenge. The global rise in ND cases underscores the vulnerability of a large population facing limited access to proper diagnosis and treatment. Growing evidence suggests that neurodegeneration is partly caused by environmental factors, specifically through neuroinflammation [1]. The buildup of protein clumps, from single molecules to fiber-like structures, makes these diseases harder to spot early on [2]. Fibril structure, function, and stability are heavily influenced by the amino acid sequence of the protein and surrounding environmental conditions [2]. Pathological markers, such as amyloid-beta and tau in Alzheimer’s, alpha-synuclein in Parkinson’s, and polyglutamine in Huntington’s, pose a major challenge to the development of effective diagnostic and treatment methods for neurological disorders.

**Figure 1:**
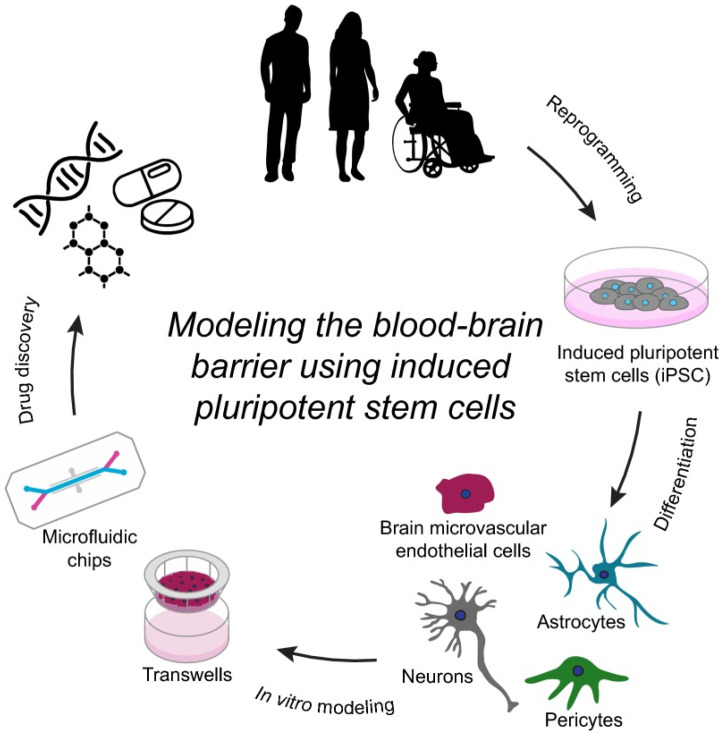
A summary of how induced pluripotent stem cells are used to model the blood–brain barrier. This figure was adapted from reference [5] with permission.

The failure of ND therapy is often attributed to the poor permeability of therapeutics across the blood–brain barrier (BBB) [3,4]. Dendrimer-based approaches show great potential in diagnosing and treating brain tumors by targeting molecular cargoes to tumor sites and efficiently crossing the blood–brain barrier after systemic administration [3]. Currently, dendrimers such as PAMAM, PPI, and PLL have been confirmed as groundbreaking for the treatment and diagnosis of brain tumors and other cancers [3]. The BBB, which consists of pericytes, astrocytes, capillary basement membranes, and tightly packed endothelial cells, plays a vital role in protecting the central nervous system (CNS) from circulating blood [5,6]. The future success of neurotherapeutics relies on advancements in drugs targeting the CNS [4]. Some of the blood–brain barrier’s main functions are to prevent the entry of dangerous chemicals into the bloodstream, maintain the brain’s internal environment, and protect it from harmful stimuli. Despite blocking harmful chemicals, the blood–brain barrier also limits the entry of therapeutic medications, decreasing drug accessibility and diminishing therapeutic advantages [7]. In vitro modeling of the human BBB has significantly progressed with the use of iPSC-derived BMECs (iBMECs), providing valuable insights into its development and function, and aiding in CNS drug discovery (Figure 1).

**Figure 2:**
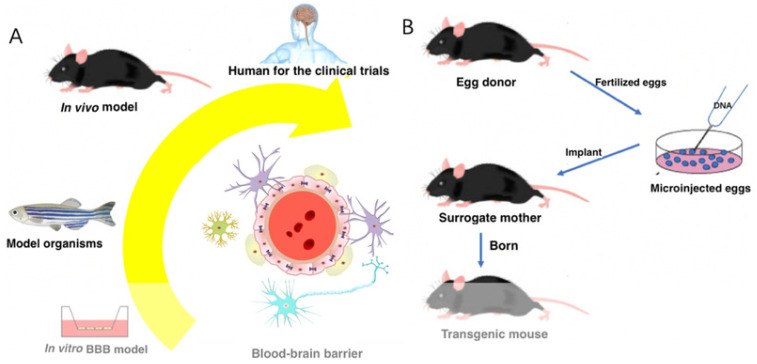
(**A**) Models employed to examine nanocarriers’ passage across the BBB. (**B**) Steps to construct in vivo BBB models. This figure was adapted from reference [9] with permission.

Nanotechnology has shown remarkable treatment effects in neurodegenerative disorders [8,9]. Inorganic NMs, polymeric NMs, micelles, liposomes, and nanofibers are among the nanomaterials designed as theranostics, showcasing numerous advantages like high drug loading, controlled drug release, precise targeting, stability, biodegradability, biocompatibility, and low toxicity [9]. Various strategies utilizing nanotechnologies were extensively employed for the transport of theranostic agents across the BBB, such as receptor-mediated transcytosis, BBB disruption through mechanical or ultrasound means, BBB crossing via a shutter peptide, and nanomaterial delivery through the intranasal route [9]. The distinguishing features of nanomaterials include small size, high surface area, and enhanced properties. Nanomaterials have diameters ranging from 1 to 100 nm, as per American Society for Testing and Materials (ASTM) and International Organization for Standardization (ISO) standards. Functionalized nanomaterials have successfully breached the blood–brain barrier through their size effects or active targeting. Nanomaterials have facilitated drug uptake through receptor-mediated endocytosis with ligands attached to their surface [10,11]. Understanding the transportability and fundamental mechanisms of nanomaterials requires the development of diverse study methods. Different study methods exhibit specific roles and characteristics depending on the level. By utilizing in vitro BBB models, high-throughput screening can be performed at a low cost, while in vivo models contribute to the clinical translation evaluation process. Figure 2 provides a brief summary and discussion of study methods for examining nanomaterials’ transportability across the BBB.

### 1.1. Parkinson’s Disease

Parkinson’s disease ranks as the second most common degenerative disease globally, with a global increase of 74.3% between 1990 and 2016 [12]. The death of mesencephalic dopaminergic (mDA) neurons in the brain’s substantia nigra pars compacta is the cause of reduced dopamine levels and subsequent neurodegeneration [13]. The NAC region of αSyn, located in the hydrophobic center, encodes the crucial segment for toxic αSyn aggregation [13].

Dysfunctional mDA neurons transform into α-synuclein-rich Lewy bodies and Lewy neuritis. α-synuclein is made up of 140 amino acids and can be divided into three domains. The N terminal domain (amino acid residue 1–60) is an amphipathic domain that interacts with the phospholipid membrane [14]. The nonamyloid β component (NAC) domain in the Rienstra group’s model is made up of six β-strands with five turns connecting them. Thus, it is clear that these three groups imply distinct folding states when considering only the NAC domain of the fibrillar structure [14]. α-synuclein mutations disrupt multiple intracellular signal programs, posing a danger to dopaminergic neurons. In transgenic mice, the A53T mutation in α-synuclein could reduce brain autophagy and lead to synucleinopathy [15]. Furthermore, A53T induces cell death pathways in PC12 cells through endoplasmic reticulum stress and mitochondrial dysfunction [16]. Overexpression of mutant α-synuclein significantly hampers proteasomal protein cleavage in dopaminergic cells such as SH-SY5Y and PC12 [17]. Matsumoto and colleagues proposed a novel method in which α-synuclein-carrying extracellular vesicles derived from erythrocytes can traverse the blood–brain barrier, facilitating communication between the brain and the periphery in the development and advancement of Parkinson’s disease [18].

### 1.2. Alzheimer’s Disease

**Figure 3:**
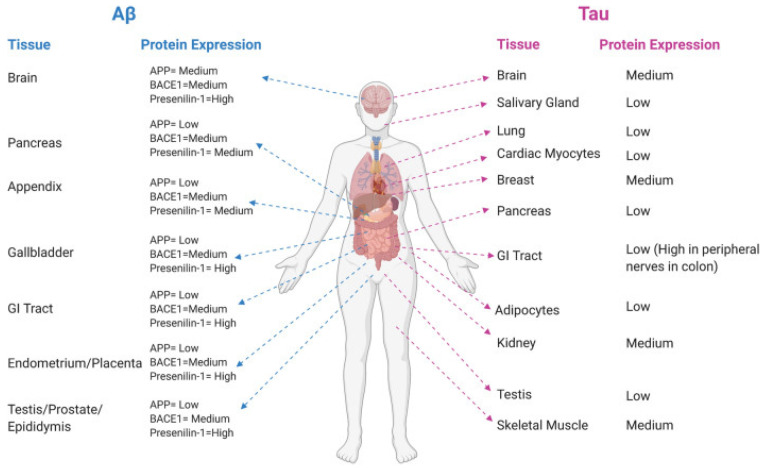
The Human Protein Atlas provides information on the tissue-level protein expression of APP, BACE1, presenilin-1, and tau, indicating potential for Aβ production. This figure was adapted from reference [20] with permission.

Globally, more than 50 million individuals are affected by AD, making it the most prevalent neurodegenerative disorder [19]. Both health disparities and health care disparities continue to exist in the US, despite efforts to promote equity [19]. New findings from surveys commissioned by the Alzheimer’s Association shed light on discrimination’s role in dementia care, disparities in trust within medical research, and variations in awareness of and concern about Alzheimer’s among different racial and ethnic groups [19]. The Human Protein Atlas reported that tau and the essential proteins for Aβ production are expressed in various body tissues (Figure 3). The hallmarks of this disorder include the development of intracellular neurofibrillary tangles (NFTs) with tau proteins, extracellular senile plaques with amyloid β (Aβ) peptides, synapse loss, and neuronal death. The main indication of AD is the progressive development of dementia [20]. The identification of microtubule-associated protein tau (MAPT) and Aβ as the components of NFTs and extracellular plaques, respectively, has spurred extensive study on the harmful effects of these proteins in the development of AD [21,22,23]. The APP gene on chromosome 2 encodes for amyloid precursor protein (APP) [24]. The series of cleavages of APP by beta-site amyloid precursor protein cleaving enzyme 1 (BACE1) [25] and γ-secretase [26] lead to the production of Aβ. Recent evidence suggests that it forms a complex with multiple proteases to maximize cleavage efficiency [27].

APP is cleaved by γ-secretase and β-secretase, leading to the formation of the Aβ42 monomer, which tends to aggregate abnormally [28]. Cu^2+^ is involved in the pathology of AD, playing a complex role in the generation of reactive oxygen species (ROS) and the promotion of Aβ lesions [29]. Research has shown that the Aβ42 monomer creates Aβ oligomers that cause neurotoxic effects through various pathways. At first, Aβ oligomers trigger N methyl D aspartate receptor (NMDAR) activation on neural membranes, causing ER stress and neuronal apoptosis [30]. Additionally, Aβ oligomers cause cell membrane harm by puncturing the lipid bilayer and creating fibrils [31]. Aβ oligomers trigger mitochondrial depolarization, leading to mitochondrial stress and excessive ROS production [32]. Excessive metal ions in the brain microenvironment, which are linked to AD pathophysiology, generate ROS via Fenton/Fenton-like reactions and decrease glutathione levels, thereby exacerbating mitochondrial stress [33]. AD studies also indicate that mitochondrial oxidative stress is its main cause. Aβ oligomers can cause leakage in endosomal/lysosomal membranes, leading to neuronal apoptosis [34].

### 1.3. Tauopathy and Autophagy

Tauopathy is a defining characteristic of AD, marked by abnormal accumulation of tau protein. Post-translational modifications (PTMs) such as phosphorylation, acetylation, and glycosylation regulate the physiological function of Tau [35]. Under normal physiological conditions, tau binds to tubulin to regulate cytoskeletal homeostasis. However, abnormal PTMs are induced by Zn^2+^ and Fe^2+^ in AD, leading to the hyperphosphorylation of tau [36]. As a result, its ability to bind to tubulin weakens. Additionally, tau hyperphosphorylation enhances self-binding, resulting in the formation of tau oligomers and more advanced NFTs [28]. Tau’s abnormal behavior leads to changes in the cytoskeleton, impaired synaptic function, and ER stress. Recent evidence indicates that Aβ can activate GSK-3β, leading to tau hyperphosphorylation, while p-tau can promote Aβ cleavage [37,38]. New research has indicated an intricate connection between tauopathy and microglia. Blocking the NLRP3 inflammasome pathway in microglia can alleviate tauopathy [39].

Three major types of autophagy have been reported in cells. The types of autophagy include microautophagy, chaperone-mediated autophagy, and macroautophagy [40]. During microautophagy, lysosomes directly engulf small protein complexes by invaginating their own membrane. The role of microautophagy in brain synapse maintenance has been suggested, but more research is needed to understand its impact on AD [41].

Treating proteinopathies like AD with the selective degradation of misfolded proteins seems promising. However, more research is needed to determine the impact of CMA deficiencies in the development of AD and to identify potential therapeutic targets in this pathway [42].

### 1.4. Huntington’s (HTT) Disease

Huntington’s disease results from a cytosine–adenine–guanine (CAG) extension in the HTT gene, leading to neurodegeneration and symptoms like motor dysfunction, cognitive disability, and psychiatric disturbance [43]. Early investigations of HTT striatal disorders indicated a decline in GABAergic and cholinergic neurons, while dopaminergic terminals were relatively unaffected [43]. HTT’s size and flexibility enable it to interact with other proteins, playing various roles in cellular processes [44]. The polyglutamine (polyQ) region of HTT alters the protein’s structure and protein–protein interactions, potentially impacting cellular processes [45].

HTT has a crucial role in autophagy through its interactions with p62 and ULK1 [46]. HTT inhibits the ULK1/2 complex by competing with mTOR for ULK1, and it can induce autophagy by releasing ULK1 from mTORC1. HTT also interacts with p62 to transport ubiquitinated substrates to an autophagosome. It has been shown that when polyQ is removed from HTT, neuronal autophagy increases [47]. The conformational alteration of HTT due to polyQ expansion can inhibit autophagic pathways in neurons, thereby leading to neurodegeneration.

## 2. Implication of Blood–Brain Barrier

The brain, crucial for metabolism and coordinating overall activity, is the body’s most vital organ. The “barrier” between the central nervous system (CNS) and the rest of the body is an interface that selectively keeps most things out and regulates the CNS microenvironment.

The BBB is composed of three cellular elements of brain microvasculature [2], viz. the inner layer which consists of cerebral micro-vessel endothelial cells (CMECs) and tight junctions [48], the middle which consists of pericytes and middle lamella [49], and the outer layer which consists of basement membrane (rich in proteoglycans, namely heparin sulfate and proteins such as collagen type IV and laminin) and astrocyte end feet [50].

In summary, pericytes are versatile cells that surround endothelial cells and regulate junctions and transcytosis in the blood–brain barrier.

The controlled passage of NPs through the BBB is made possible by the intricate architecture of this membrane. Astrocytes and glial cells play a role in clearing debris and maintaining ion balance in the cerebrospinal fluid. Microglia, the primary macrophage cells in the brain, help eliminate protein aggregates and become active in response to trauma and systemic inflammation. NPs cross the BBB through three main pathways: paracellular, transcellular, and carrier-mediated. NPs migrate through the paracellular pathway due to concentration gradients [51]. Conversely, transcellular transportation relies on receptor-mediated active transport and ATP energy expenditure [52]. However, most NPs cross the blood–brain barrier through a substrate-specific pathway known as the endogenous transporter or carrier-mediated pathway, such as carbon dots and other organic and inorganic NMs combined with medicinal molecules. NPs can select GLUT 1, LAT 1, Angiopep 2, and Seq 12 receptors to enhance their transit across the BBB and improve therapeutic effectiveness [53].

## 3. Nanomaterials for Targeted Therapy of Neurodegenerative Diseases (NDDs)

NDDs negatively affect the living conditions of the elderly, especially those over fifty. Meeting the urgent medical demand of addressing NDDs requires the development of new treatments. Although they have varying histopathological aspects, these illnesses share common cellular traits and disease-progression signaling pathways [23]. Despite the distinct protein aggregates observed in each neurodegenerative disorder, there are also instances where multiple pathological proteins aggregate. Transgenic animal and cell culture studies indicate various interactions between neurodegeneration-related proteins, like aggregate cross-seeding and protein mislocalization, contributing to disease progression [23]. Protein precipitates or aggregates are often seen in the extracellular matrix or apoptotic neurons in the nervous system’s NDD-related brain regions [54]. The specific way in which protein misfolding and aggregation contribute to neurodegeneration is not known, but three broad models can be proposed: the loss of normal protein activity, the acquisition of neurotoxicity through misfolding, and chronic brain inflammation triggered by the accumulation of protein deposits [54]. Researchers are actively investigating various methods to hinder and potentially reverse protein misfolding and aggregation, in the hopes of developing effective drugs for neurodegenerative disorders [54]. Most of these aggregates are made up of denatured pathogenic proteins, as shown by researchers. Advances in nanomedicine have simplified the elimination of these harmful proteins by hindering the onset and spread of disease. Lowering the amount of toxic Aβ aggregates is a common strategy for addressing AD.

### 3.1. The Potential of siRNA in Treating Neurodegenerative Disease

Inhibiting BACE1, which is necessary for breaking down Aβ precursors, has proven to be beneficial in treating AD. As an example, Singer et al. utilized siRNAs to inhibit Aβ synthesis [55]. RNA interference (RNAi) therapeutics are being actively researched for targeted gene suppression and are currently undergoing clinical trials. Selectively silencing the key players involved in the AD pathway is a potential benefit of RNAi. To unlock the full potential of RNA therapeutics like siRNAs, customized cationic carriers can be used to package these siRNAs into NMs or complexes. This packaging not only safeguards the RNA therapeutics but also aids in delivering the NMs to target cells [56]. Despite the potential of siRNAs in AD treatment, there are significant challenges in delivering them locally and systemically. Developing nano-carriers with distinct advantages is essential for siRNA transportation. Gene therapy applications have shown that Linear polyethylenimine (LPEI) is a versatile carrier. A micellar nanoparticle system utilizing the LPEI-g-PEG copolymer showed remarkable siRNA distribution ability [56]. By injecting LPEI-g-PEG NMs containing siRNA into the lateral ventricles of mice, BACE1 was effectively suppressed in the brain. Due to the BBB’s limited permeability and the low presence of neurons expressing high levels of BACE1, it is generally not recommended to introduce siRNA systemically into the central nervous system. Furthermore, the circulation of siRNA nanocomplexes in the blood could potentially trigger inflammatory or immunological responses. To address these problems, Wang et al. developed a neuron-targeted nanocomplex by incorporating Tet1 and CGN peptides into PEGylated poly (2-(N,N dimethylamino) ethyl methacrylate) (PEG-PDMAEMA). This allowed for the specific targeting of neurons and penetration through the blood–brain barrier (BBB). The nanocomplex effectively reduced the BACE1 mRNA level to about 50%, resulting in the improvement of cognitive impairment in AD transgenic mice [57]. The fibrillation of Aβ can be regulated by certain sequences that impact the early conformational transition and oligomerization of Aβ. By using synthetic peptide inhibitors to disrupt these important amyloidogenic areas and produce benign hetero oligomers, dangerous Aβ fibrillation is effectively prevented. Nonetheless, peptide inhibitors can break down inside the body and tend to self-assemble. Xiong et al. [58] combined Soto’s β-sheet breaker peptide (LPFFD) with two sequences (Aβ39-42, VVIA) into a single chain and investigated how this impacted Aβ42 aggregation and cytotoxicity. However, when the peptides are conjugated onto gold NMs (AuNPs), inhibition activity against Aβ42 aggregation and cytotoxicity is significantly enhanced. The delivery technique of intraventricular infusions of LPEI/siRNA NMs and shaped micellar NMs in awake, freely moving mice aims to achieve the widespread distribution of the payload in the brain [56].

### 3.2. A Nanomaterials Strategy for Overcoming Barriers

The high surface-to-mass ratio, stability, and hydrophilic/hydrophobic affinity of NMs make them perfect for treating NDs. NPs have the ability to imitate lipidic cell membranes by encapsulating hydrophilic and hydrophobic substances. Various molecules can be used to functionalize them for biosensing and drug delivery [59]. Active transport is involved in endocytosis, the primary internalization process. The energy required for internalization relies on the nanosystem’s size, surface charge, and flexibility [60]. The highest energy demand is for silica and metal NMs. Lipid NMs adhere to cell membranes through hydrophobic interactions, allowing for easier fusion and uptake compared to silica and metal NMs with more rigid structures [61]. On the other hand, a positive charge on NPs improves their interaction and uptake in diseased tissues, thanks to the enhanced permeability and retention (EPR) effect. Stimuli-responsive nanocarriers are extensively utilized to enhance intracellular drug release by delivering therapeutics in response to biological stimuli like ROS, pH, and temperature.

Membrane gaps allow the passive internalization of hydrophobic NPs, while protein channels and carriers facilitate the entry of hydrophilic NPs. NP uptake mechanisms were extensively described by Sabourian et al. Medication release can be improved by nanocarriers that respond to biological stimuli such as pH, temperature, or ROS [62]. pH-sensitive NMs are utilized to treat tumors and their microenvironment, as changes in pH play a vital role in lesion development, and can be used to release medications based on pH. NMs’ adjustable properties improve pharmacokinetics and address challenges in treating neurodegenerative diseases.

These challenges involve accessing the desired tissue or compartment without causing harm locally or systemically. The bioavailability of small-molecule medicines can be reduced at the intended treatment site due to hepatic metabolism and clearance. The dose of the medicine given is higher than what is effective, which can cause accumulation and potentially harmful side effects. Drug bioavailability can be improved, dosages reduced, and retention durations extended with nanosystems. Nanoemulsions and nanocrystals have experienced an enhancement of their properties due to recent advancements in nanomaterials.

Nanoemulsions are tiny emulsions, typically between 20 and 200 nm, in which two liquids that do not normally mix are combined with an emulsifier to form a single phase. Nanoemulsions are smaller than regular emulsions, allowing for easier administration, absorption, and engraftment. On the other hand, nanocrystals are solid carriers that are usually negatively charged and have amorphous and lipophilic surfaces. The purpose of these systems is to enhance the delivery of active medicinal ingredients. Studies have demonstrated that nanoemulsions are more efficient in delivering chemicals to the CNS [63] compared to the intravenous administration of small-molecule drugs [64]. In animal models of Alzheimer’s, multiple sclerosis, and Parkinson’s disease, preclinical studies revealed enhanced neurorepair and neuronal resistance with gold nanocrystal suspensions. Following successful phase I trials, the treatment of gold nanocrystals (CNM-Au8) was approved for phase II clinical trials in early ALS patients [65].

Neurodegeneration is primarily caused by neuroinflammation. Microglial cells in the CNS start the inflammatory response, resulting in the release of pro-inflammatory cytokines in reaction to harmful substances [66]. Recent evidence shows that NMs are a successful treatment for neuroinflammation and protein aggregation. AD animal models have shown reduced neuroinflammation and Aβ aggregation with liposomes containing phosphatidic acid, cardiolipin, immune-PEG, and PLGA. The study discovered that lipidic nanocarriers, when injected intraperitoneally, can reach the lymphatic system and bloodstream. The strong attraction and attachment of liposomes led to the elimination of free Aβ from the CNS, also known as the “sink effect” [67].

**Figure 4:**
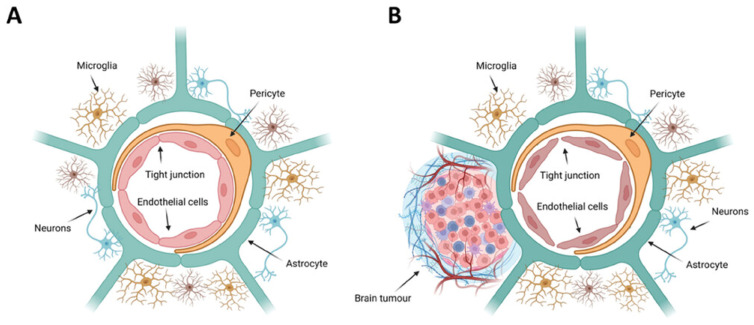
The biological composition of (**A**) the blood–brain barrier (BBB) and (**B**) the blood–brain tumor barrier (BBTB). This figure was adapted from reference [69] with permission.

PEG and PLGA, biocompatible polymers, create a steric barrier for nanocarriers to avoid opsonization and increase their retention in the bloodstream. PEI-loaded NMs have shown similar results, suggesting PEI’s effectiveness in delivering different compounds [68]. AuNPs can effectively reduce neuroinflammation and protein aggregation formation by stabilizing chiral peptide inhibitors. The use of viruses to express siRNA has its drawbacks, such as limited effect at the injection site and challenges in controlling RNAi dosage response. The exploration of RNAi-based therapeutic approaches in the brain through siRNA delivery has been limited due to the poor transfectability of neuronal cells. Efficient RNAi requires the successful penetration of therapeutically active siRNA into target cells [68] (Figure 4).

### 3.3. Barrier Between Blood and Brain Tumors

The delivery of bioactive agents to the brain can be achieved through alternative pathways. For instance, intranasal delivery is an innovative method that bypasses the BBB, leading to advantages like enhanced brain drug delivery, lower systemic exposure, and fewer side effects. While nasal delivery of NMs holds promise for improved brain drug delivery, challenges like mucociliary clearance and pulmonary effects must be addressed for broader adoption [69]. The BBB is crucial in keeping harmful substances out of the brain, as shown in Figure 4.

Key cellular antioxidant defense systems involve enzymes like superoxide dismutase, catalase, and glutathione peroxidase. These enzymes protect against damage by getting rid of ROS. Cellular damage and dysfunction, caused by oxidative stress, can result in various diseases [70]. For instance, oxidative stress in cardiovascular disease causes low-density lipoprotein cholesterol to oxidize, promoting atherosclerosis. Neuronal damage and cell death in neurodegenerative diseases, including Alzheimer’s, can be caused by oxidative stress. ROS can be a double-edged sword in cancer, promoting tumor growth while also acting as a suppressor. ROS can induce carcinogenesis through DNA damage, potentially leading to mutations and the activation of oncogenes. Conversely, ROS can hinder cancer development by triggering cell death and inhibiting the growth of cancer cells [70]. Singlet oxygen (SO), produced by photosensitizers and visible light, offers a noninvasive approach to inhibiting Aβ aggregation and toxicity, demonstrating a unique photodynamic therapeutic effect [71]. Some nanoparticle-based drugs are already on the market, while others are being tested in clinical trials (Table 1).

## 4. Conclusions

Multiple interactions between pathological proteins have been observed in human autopsy samples and experimental models, with evidence of co-aggregation and shared involvement in molecular pathways associated with neurodegeneration. The latest data raise the possibility of pathological proteins interacting as the disease spreads through the brain. To better understand protein–protein interactions in the multimorbid old brain, it is crucial to conduct large-scale studies on post-mortem brains that incorporate detailed clinical data and quantitative measures of protein-aggregate burden. To address this goal, researchers created and tested a multifunctional liposome platform that targets toxic AβOs, aiming for the early detection and treatment of Alzheimer’s disease [77]. Understanding these interactions is key in the development of effective therapeutic targets that can minimize toxicity and potentially halt the advancement of these diseases. Expanding the use of shape as a tunable parameter in siRNA delivery strategies would enhance our ability to create a versatile platform for RNAi therapeutics. Additional studies are required to definitively demonstrate the connection between protein misfolding and aggregation as a common factor in neurodegenerative diseases. Future work should also prioritize understanding how alternative protein folding contributes to other diseases and normal cellular functions.

## Figures and Tables

**Table 1:** Investigating the use of liposomes for the enhanced delivery of treatments to brain tumors by crossing the blood–brain barrier.

Product Name	Nanoparticles	Cargo	Brain Tumor	Status of Clinical Trial	Refs.
Caelyx (Schering Plough, Weimar, Germany)	Liposomes	Doxorubicin	Recurrent malignant glioma	Commercialized	[72]
Caelyx (Essex Pharma, Munich, Germany)				Commercialized	[73]
2B3-101				Phase II	[74]
CPT-11		Irinotecan		Phase I	[75]
Anti-EGFR ILs-dox		Doxorubicin		Phase I	[76]
